# Effects of Levetiracetam and Lacosamide on survival and seizure control in IDH-wild type glioblastoma during temozolomide plus radiation adjuvant therapy^☆^

**DOI:** 10.1016/j.bas.2023.102732

**Published:** 2023-12-14

**Authors:** Andrea Bianconi, Emanuele Koumantakis, Andrea Gatto, Pietro Zeppa, Ayoub Saaid, Elsa Nico, Francesco Bruno, Alessia Pellerino, Francesca Rizzo, Carola Vera Junemann, Antonio Melcarne, Diego Garbossa, Paola Dalmasso, Fabio Cofano

**Affiliations:** a Neurosurgery, Department of Neuroscience, University of Turin, Turin, Italy; b Department of Public Health and Pediatrics, University of Turin, Turin, Italy; c Post Graduate School of Medical Statistics, University of Turin, Turin, Italy; d Department of Neurosurgery, Barrow Neurological Institute, St. Joseph's Hospital and Medical Center, Phoenix, AZ, USA; e Neurooncology, Department of Neuroscience, University of Turin, Turin, Italy; f Neurology, Department of Neuroscience, University of Turin, Turin, Italy

**Keywords:** Anti-Seizure medication, Brain tumor related epilepsy, Epilepsy, Glioblastoma, Lacosamide, Levetiracetam

## Abstract

**Introduction:**

There are no clear indications for the best choice of anti-seizure medications to control brain tumor related epilepsy. In vitro studies have shown an antitumoral effect of Levetiracetam and Lacosamide on glioblastoma IDH-wild type.

**Research question:**

This study investigates whether the use of levetiracetam and/or lacosamide impacts survival rates. The secondary aim was to evaluate the efficacy of both ASMs in controlling seizures.

**Materials and methods:**

In this observational retrospective single-cohort study, patients underwent chemoradiation protocol after GBM surgery. They were grouped as follows: (1) use of levetiracetam, (2) use of lacosamide, (3) simultaneous use of levetiracetam and lacosamide, (4) no ASM usage. Survival curves were plotted using the Kaplan-Meier method coupled with a log-rank test for difference assesments. To evaluate the pharmacological efficacy of post-operative seizure control, a negative binomial regression was conducted.

**Results:**

The study included 272 patients, 174 of which underwent adjuvant chemoradiation treatment. Patients without ASM therapy had a non-significant longer median OS (compared to the other groups (log-rank = 0.37). The IRR of seizure relapse was 2.57 (p = 0.007) times higher in lacosamide users, and MGMT promoter methylation demonstrated a protective effect against postoperative seizure onset (p = 0.05), regardless of the aforementioned confounding factors.

**Discussion and conclusions:**

In patients diagnosed with GBM IDH-WT undergoing chemoradiation therapy, the use of levetiracetam or lacosamide for controlling BTRE does not seem to modify survival. Lacosamide users exhibited a higher IRR of postoperative seizures compared to levetiracetam users, and MGMT promoter methylation appears to be a protective factor.

## Highlights

• No clear indication on best antiseizure medication in brain tumor related epilepsy, levetiracetam and lacosamide are the most commonly used.
• Single or combined therapy with Levetiracetam or Lacosamide does not seem to improve survival in vivo.

## 1 Introduction

Glioblastoma (GBM) is the most common and fatal form of malignant tumors in the central nervous system (CNS), accounting for nearly 60% of gliomas and having a 5-year survival rate of around 7% (Bianconi et al., 2022; Tewarie et al., 2021; Stupp et al., 2005).

The optimal therapeutic strategy involves gross total resection (GTR) with concomitant radiochemotherapy using Temozolomide (TMZ), (Stupp et al., 2005) and various strategies have been developed in order to maximize the extent of resection (EOR) (De Marco et al., 2022; Zeppa et al., 2022; Trifiletti et al., 2017; Brown et al., 2016). However, despite these efforts, the median overall survival (OS) and median progression-free survival (PFS) for GBM patients remain poor (Bruno et al., 2022; Suchorska et al., 2016; Saaid et al., 2022).

One common concern in the clinical management of patients with GBM is the occurrence of brain tumor-related epilepsy (BTRE). (Avila and Graber, 2010), (Vecht et al., 2014) Between 29 and 49% of patients experience at least one seizure event, which can either be the first manifestation of the disease or develop during its progression (Shin et al., 2017). BTRE significantly affects the quality of life of patients and requires effective treatment, usually with a single anti-seizure medication (ASM), to prevent further episodes (Toledo et al., 2015). However, despite the wide range of available ASMs, no definitive evidence exists regarding the superiority of one drug over the others, (Sirven et al., 2004; Kerrigan and Grant, 2011; Vecht et al., 2017), although comparisons among multiple ASMs are available, in particular, levetiracetam has shown superiority over valproic acid specifically in the treatment of glioma BTRE (van der Meer et al., 2021).

ASMs that have little or no interference with the metabolism of antineoplastic drugs are commonly administered, especially those that do not induce or inhibit liver enzymes (CYP system), such as Levetiracetam (LEV) or Lacosamide (LAC). These enzymes are responsible for metabolizing chemotherapy drugs, including TMZ, so using ASMs that do not affect their activity is preferred to avoid reducing the antitumor efficacy or increasing chemotherapy toxicity.

Many studies have investigated the antineoplastic activity of non-chemotherapy drugs (BIANCONI et al., 2023), and in vitro studies have demonstrated an antitumoral effect of LEV and LAC on GBM cells (Rizzo et al., 2017). Some clinical studies have confirmed the benefits of LEV in increasing OS for patients with IDH-wild type (IDH-WT) GBM when used in conjunction with TMZ chemotherapy after surgical resection (Roh et al., 2020; Pallud et al., 2022; Kim et al., 2015; Happold et al., 2016). On the other hand, no clinical studies have evaluated the effects of LAC on survival during adjuvant GBM therapies. As of the present day, LEV is still the most widely used ASM in BTRE, and LAC is one of the third-generation drugs recommended for monotherapy as first-line treatment in BTRE (Avila et al., 2023). Therefore, the main objective of this study, the first to compare these two ASMs, is to analyze the survival outcomes of patients with surgically treated GBM IDH-WT taking LEV and/or LAC during adjuvant chemoradiation therapy with TMZ. Additionally, the study aims to assess the effectiveness and tolerability of LEV and LAC during the postoperative period, irrespective of any adjuvant therapies.

## 2 Materials and methods

**Fig. 1:**
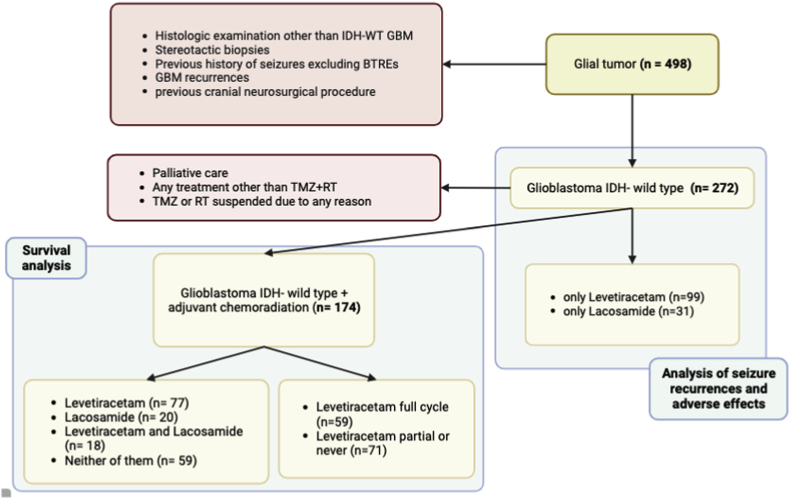
Descriptive diagram of inclusion criteria and patient selection.

An observational, retrospective single-center study was conducted, including adult patients (≥18 years old) who underwent surgical treatment of GBM IDH-WT. Histopathological and molecular diagnosis data were collected and updated according to the WHO 2021 CNS tumor classification (Louis et al., 2021). The following exclusion criteria were applied: a history of seizures apart from BTRE, previous cranial neurosurgical procedures, GBM recurrences, and stereotactic biopsies. For the survival analysis, additional exclusion criteria were considered, such as patients who received palliative care or adjuvant therapies other than concomitant chemoradiotherapy with TMZ-based regimens. Patients who had radiation therapy or TMZ suspended due to adverse effects during the course of disease were also excluded. To minimize confounding variables, patients taking any ASM other than LEV or LAC were excluded (Fig. 1).

### 2.1 Data collection

Clinical and radiological data were collected from intraoperative reports, pre and postoperative MRI scans, and outpatient reports. Preoperative data included the history of epileptic events, seizure type, documented either clinically or through EEG, and information on the type and dosage of ASMs. Postoperative data included contrast-enhanced MRI evaluation of the extent of resection (EOR) categorized as biopsy, subtotal resection (STR), gross total resection (GTR), or supra maximal resection (SMR), as well as progression-free survival (PFS) and overall survival (OS) (in months; according to RANO criteria for disease progression) (Wen et al., 2010). Adjuvant therapy data for radiotherapy (type, Gy dose, duration of treatment) and chemotherapy (specifically TMZ protocols; dose and duration of treatment) were recorded. Seizure control parameters, including the occurrence of postoperative seizures, the number of seizure recurrences, the number of months from surgery to the occurrence of the first seizure or seizure recurrence (seizure-free interval), and adverse drug reactions were also documented.

Patients were grouped based on their use of LEV and/or LAC during the chemoradiation cycle. Four groups were identified: (1) LEV only, (2) LAC only, (3) LEV and LAC simultaneously, (4) No ASM.

Given that LEV is the most widely used and researched ASM in BTRE, both in vitro and in vivo, an additional analysis was conducted to investigate the effect on OS by comparing patients who used LEV throughout the chemoradiation cycle (LEV full-time) with those who used it for a portion of the cycle (LEV part-time) or never.

Radiotherapy was initiated approximately one month after the date of surgery. Both standard (60 Gy in 30 doses) and hypofractionated (42 Gy in 14 doses) modalities were taken into account. The total duration of radiochemotherapy treatment varied depending on the radiotherapy protocol; it was either 3 weeks or 6 weeks. Afterwards, patients received cycles of TMZ alone for either 6 months or 12 months. The duration of TMZ treatment was determined based on the clinical status or imaging changes of the patients. (Stupp et al., 2005)., (Perry et al., 2017)

Administration of ASMs was initiated before surgery in the case of a preoperative seizure event. If a seizure event occurred during follow-up, ASMs were started at that time. No prophylactic ASMs were used before the neurosurgical procedure. In order for a patient to be considered on LEV or LAC therapy, the dosage had to fall within the following therapeutic ranges: LEV dosage between 1000 mg and 2500 mg daily and LAC dosage between 100 and 200 mg daily. Furthermore, at least one monitoring of serum concentrations had to confirm that the drug levels were within the therapeutic range (12–46 mg/L for LEV and 2–20 mg/L for LAC). The presence of kidney dysfunction or liver disease was noted, and any changes in therapy were documented. The duration of treatment with ASMs during adjuvant radiochemotherapy was recorded and expressed in months.

### 2.2 Statistical analysis

Survival analysis was carried out using the Kaplan-Meier estimator. Death was considered as a failure event, and the observation period was set from the months following surgery since January 1, 2015 until December 31, 2021. The survival functions were estimated for (i) the entire sample, (ii) patient groups based on the ASM treatment received in the postoperative period (LEV, LAC, both, or neither), and (iii) patient groups based on the duration of LEV treatment (full-time vs. part-time or never). In the last two analyses, the logarithmic ranks test (log-rank test) was used to compare the survival distribution between groups.

To compare the effectiveness of the LEV and LAC regimens between groups, the number of epileptic events after the surgery date was considered as the outcome variable. Since the dependent variable is a count, an overdispersion test was conducted to determine whether a Poisson regression model or a negative binomial regression model should be used. If overdispersion was significant, a negative binomial regression model was preferred; otherwise, a Poisson regression model was used. Regardless of the choice of the regression model, the type of ASM used (LAC only or LEV only) was the main independent variable. Covariates such as MGMT methylation, preoperative seizures, and lesion site, which are known risk factors for seizures in the postoperative course, were included in the model, along with sex and age.

For all analyses, α = 0.05 was set as the significance level. STATA (SE 17.0) was used as the statistical software.

## 3 Results

### 3.1 Patients characteristics

The study enrolled 272 patients who underwent resection of GBM IDH-WT. Table 1 shows the sample characteristics. Among the patients, 88 (32%) experienced seizures before surgery. The most common type of seizures observed were generalized seizures (43 patients) followed by focal seizures (38 patients). Only a small percentage (2.6%) of patients experienced focal seizures with secondary generalization. EOR was GTR in 207 patients, STR in 30, supramaximal in 17, and open biopsy in 15. Molecular data showed that the MGMT promoter was methylated in 101 patients (44%). A total of 186 (69%) patients underwent postoperative radiotherapy, while 199 (73%) patients received chemotherapy with TMZ. According to the Stupp or Perry protocol, a total of 174 patients received the complete cycle of concomitant radiochemotherapy.

**Table 1:** Descriptive table of sample characteristics. Data are presented as median (IQR) for continuous measures, while for categorical variables count (percentage) are provided. The percentage relative frequencies for the variables “Major types of seizures” and “Types of seizures” are calculated considering the total number of subjects with a history of seizures.

Sample Characteristics	n = 272
Sex: Male	167 (61.4%)
Age (years)	63 (55–71)
Tumor site	
Pre-central	110 (40.8%)
Post-central	56 (20.6%)
Temporo-insular	94 (34.6%)
Basal ganglia/midbrain/multicentric	12 (4.0%)
History of seizures: Yes	88 (32.4%)
Major types of seizures	
Focal	38 (43.2%)
Generalized	43 (48.9%)
Secondary generalization	7 (8.0%)
Types of seizures	
Aphasic seizures	6 (6.8%)
Absences	2 (2.3%)
Motor seizures	60 (68.2%)
Neurovegetative seizures	2 (2.3%)
Sensitive seizures	4 (4.5%)
Visually sensitive seizures	5 (5.7%)
Uncinate seizures	9 (10.2%)
MGMT promoter methylation: Yes	101 (37.1%)
Type of surgery	
Supramaximal resection	17 (6.6%)
Gross total resection	196 (76.0%)
Subtotal resection	30 (11.6%)
Biopsy	15 (5.8%)
Adjuvant radiotherapy: Yes	186 (68.6%)
Adjuvant temozolomide: Yes	199 (73.4%)
Use of Levetiracetam/Lacosamide	
Levetiracetam	99 (36.4%)
Lacosamide	31 (11.4%)
Both of them	35 (12.9%)
Neither of them	107 (39.3%)
Seizures after surgery	
None	170 (62.5%)
One	65 (23.9%)
Two or more	37 (13.6%)

### 3.2 Survival analysis

The median OS of our cohort (n = 272) was 15.64 months.

**Fig. 2:**
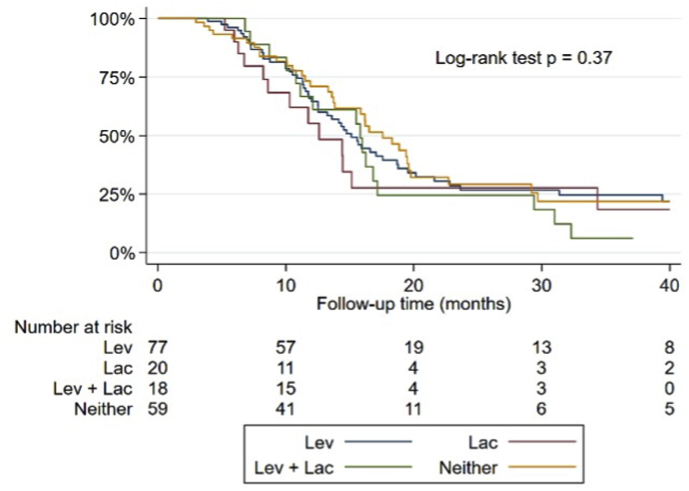
Kaplan-Meier survival curves for patients who took only levetiracetam (blue line), only lacosamide (red line), both levetiracetam and lacosamide (green line), and neither of the two drugs (orange line). For comparison between the curves, log-rank test's p-value is reported. Lev = levetiracetam, Lac = lacosamide.

To assess the effects of LEV and LAC on survival, only patients who underwent the chemoradiotherapy protocol according to Stupp or Perry were included (n = 174). Among these patients, 77 received only LEV therapy (LEV group), 20 received only LAC therapy (LAC group), 18 received both medications simultaneously (LEV + LAC group), and 59 did not receive either of these medications (NULL group). When comparing the survival curves, there were no clear differences in trends between the groups (Fig. 2). In fact, the comparison of survival probabilities using the log-rank test was not statistically significant (p = 0.37).

The median survival time estimates varied among the groups. The NULL group had the highest median survival time estimate of 17.55 months. Conversely, the LAC group had the lowest median survival time estimate of 12.58 months. The LEV group and the LEV + LAC group had very similar median survival time estimates of 15.10 months and 15.80 months, respectively.

**Fig. 3:**
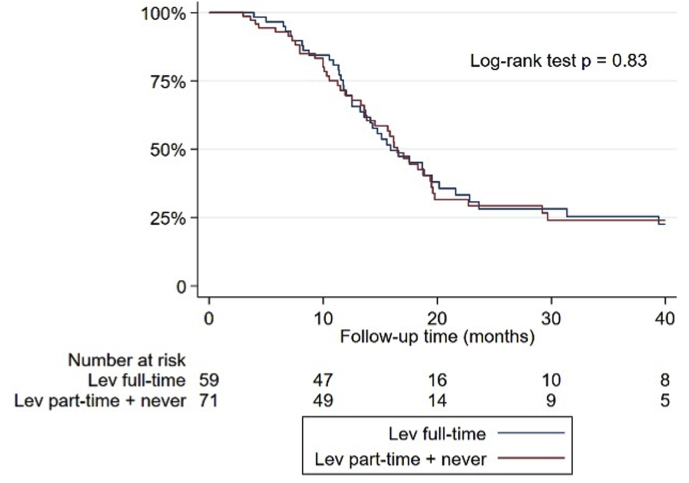
Kaplan-Meier survival curves for patients who took only levetiracetam full-time (blue line) or part-time/never (red line). For comparison between the two curves, log-rank test's p-value is reported. Lev = levetiracetam.

In order to examine the potential impact of LEV use on survival, patients who exclusively received LEV during the radiochemotherapy period with TMZ were selected. The aim was to assess whether the duration of LEV intake, compared to the length of chemoradiotherapy, had any effect on survival. Two groups were compared: (1) patients who took LEV from the start of radiochemotherapy (LEV full-time) and (2) patients who started taking LEV after the initiation of radiochemotherapy (LEV part-time) or who did not use any ASMs during the same period. A total of 130 patients were included in the analysis, with 59 in the LEV full-time group and 71 in the LEV part-time or never group. The survival curves of the two groups were comparable, with median survival time estimates of 15.9 months and 16.5 months in the first and second groups, respectively (Fig. 3). The log-rank test, however, was not statistically significant (p = 0.83). Thus, the intake of LEV from the beginning of radiochemotherapy with TMZ, as well as its later initiation or non-use during this period, did not have a significant impact on survival.

### 3.3 Efficacy and adverse effects

Of the 272 enrolled patients, 170 had no seizures after surgery, while 102 had at least one seizure event. Among those, 65 patients had one seizure event and 37 patients had two or more events. Of note, 44 patients had BTRE before surgery, so 50 percent of patients with epileptogenic tumors experienced at least one seizure during follow-up. None of the patients had renal or liver disease. To eliminate the confounding effect of other ASMs or combination therapy with LEV and LAC on seizure control, only patients taking LEV alone (n = 99) and LAC alone (n = 31) were included for further analysis.

Since the overdispersion test yielded a significant result (p < 0.001), as shown in Table 2, a negative binomial regression model was used to evaluate the association between the use of LEC or LAV and the recurrence of seizures in the postoperative period.

**Table 2:** Univariate negative binomial regression model's effects. Seizures recurrence Incidence Rate Ratios (IRRs), 95% Confidence Intervals (CI), and p-values are provided. Independent variable was treatment group. ASM = antiseizure medication.

	Seizures recurrences		
Predictors	IRR	95%CI	p
ASM used (reference: only levetiracetam)			
Only lacosamide	2.77	1.55–4.95	**0.001**
Both levetiracetam and lacosamide	3.23	1.87–5.61	**<0.001**
Neither of them	0.15	0.07–0.29	**<0.001**
Observations	272		

The analysis revealed that taking LAC as an ASM, compared to LEV, appeared to be associated with a 2.66-fold higher incidence rate of seizure recurrence (IRR: 2.66; 95% CI: 1.45–4.90; p = 0.002). This association remained significant regardless of the sex, age, methylation status of the MGMT gene, prior seizure history, or lesion location. Interestingly, the methylation of the MGMT gene seemed to have a protective effect (IRR: 0.55; 95% CI: 0.30–1.01; p = 0.05), even after adjusting for other covariates, while female sex increased the incidence rate of seizure recurrences (IRR: 1.79; 95% CI: 0.99–3.27; p: 0.056), even if not significantly (Table 3) (see Table 4).

**Table 3:** Multivariable negative binomial regression model's effects. Seizures recurrence Incidence Rate Ratios (IRRs), 95% Confidence Intervals (CI), and p-values are provided. Independent variable was treatment group (patients who received only lacosamide vs patients who received only levetiracetam), while adjustment was made for sex, age, history of seizures, tumor site, and MGMT methylation. ASM = antiseizure medication.

	Seizures recurrences		
Predictors	IRR	95%CI	p
ASM used: Lacosamide (vs Levetiracetam)	2.66	1.45–4.90	**0.002**
Sex: Female	1.79	0.99–3.27	0.056
Age	1.01	0.99–1.04	0.362
History of seizures: Yes	1.11	0.63–1.95	0.721
Tumor site (reference pre-central):			
Post-central	1.64	0.79–3.40	0.189
Temporo-insular	0.87	0.45–1.70	0.690
Basal ganglia/midbrain/multicentric	1.06	0.12–9.31	0.956
MGMT methylation	0.55	0.30–1.01	0.050
Observations	130

**Table 4:** Adverse effects in the study cohort. Comparisons between treatment groups (only levetiracetam vs only lacosamide groups) are performed using Fisher's exact test and corresponding p-values are reported.

Adverse effects	Overall n = 272	Levetiracetam n = 99	Lacosamide n = 31	p-value
Psychiatric:	21 (7.7%)	14 (14.1%)	3 (9.7%)	0.761
Psychomotor restlessness	10 (3.7%)	5 (5.1%)	2 (6.5%)	0.671
Alienation of thought	1 (0.4%)	1 (1.0%)	0 (0.0%)	0.999
Irritability	4 (1.5%)	3 (3.0%)	1 (3.2%)	0.999
Ideomotor slowdown	2 (0.7%)	2 (2.0%)	0 (0.0%)	0.999
Sleepiness	4 (1.5%)	3 (3.0%)	0 (0.0%)	0.999
Systemic:	5 (1.8%)	0 (0.0%)	2 (6.5%)	0.055
Aspecific Intolerance	2 (0.7%)	0 (0.0%)	0 (0.0%)	–
Epigastric pain	1 (0.4%)	0 (0.0%)	1 (3.2%)	0.238
Dermatologic reaction	1 (0.4%)	0 (0.0%)	0 (0.0%)	–
Gastrointestinal toxicity	1 (0.4%)	0 (0.0%)	1 (3.2%)	0.238

Regarding adverse effects, both drugs were generally well tolerated. In particular, 14 patients (14.1%) on LEV therapy and 3 patients (9.7%) on LAC therapy experienced psychiatric side effects. However, no statistically significant differences were observed between the two groups.

## 4 Discussion

Currently, there are no definitive guidelines regarding the optimal choice of ASMs to manage BTRE. ASMs that do not induce or inhibit hepatic enzymes, such as LEV and LAC, are generally recommended (Dewan et al., 2017).

LEV primarily works by inhibiting intraneuronal calcium currents and SV2A proteins, thereby preventing exocytosis of presynaptic vesicles and reducing neurotransmission (Sills and Rogawski, 2020). It is generally well tolerated by patients; however, it may cause neuropsychiatric adverse effects (NPAEs) including irritability, psychomotor agitation, and anxiety (Bedetti et al., 2017).

On the other hand, LAC carries out its anticonvulsant effect through the inhibition of voltage-dependent sodium channels during the slow depolarization phase. (Sills and Rogawski, 2020), (Cawello, 2015) It is known for its excellent tolerability and has demonstrated particular effectiveness in treating recurrent critical episodes when used as add-on therapy to other basic ASMs. (Saria et al., 2013), (Rudà et al., 2018) Moreover, LAC can extend the seizure-free period regardless of tumor activity and response to antineoplastic therapies (Mo et al., 2022).

### 4.1 Use of Levetiracetam and OS

The relationship between LEV and GBM survival is controversial. A pooled analysis of prospective clinical trials in new diagnosed GBM including 1869 patients found that LEV provides no survival benefit when used during the chemoradiation protocol with TMZ (Happold et al., 2016); however, other observational studies, albeit small, have shown that LEV use may be beneficial in increasing survival. (Roh et al., 2020), (Kim et al., 2015) One recently published article states that the use of LEV throughout the entire duration of the radiochemotherapy protocol with TMZ significantly increases OS (Pallud et al., 2022).

The mechanism through which LEV could increase survival is likely multifactorial. An in vitro study demonstrated that LEV can reduce the expression of MGMT by increasing the sensitivity of GBM cells to the action of TMZ, resulting in apoptosis through a mechanism dependent on p53, mSin3A and HDAC1 (Bobustuc et al., 2010). Nonetheless, this study did not demonstrate the same benefit in vivo. The in vitro study assumes that for glioma cells to undergo apoptosis, all the repressor components listed above, including p53, must either not be mutated or at least be functional (Bobustuc et al., 2010). However, this cannot be assumed in a tumor like GBM, where alterations in the proper functioning of p53, mSin3A or HDAC1 may occur. As a result, the sensitizing effect of LEV towards TMZ may not be observed, as was potentially the case in our study, due to a lack of molecular data on the mutation status of the genes mentioned above. Furthermore, the in vitro study indicated that the antitumoral effect of LEV occurs via a reduction in MGMT expression levels. It can be inferred that patients with low MGMT expression levels, based on the intrinsic molecular characteristics of the tumor, may not experience the same reduction in enzyme levels compared to patients with higher initial MGMT expression levels. Another consideration stemming from the study's results is that the reduction in MGMT levels observed in vitro may not occur in vivo. Even if the in vitro study analyzed drug concentrations equivalent to those used in clinical practice, various pharmacokinetic factors can influence the actual concentration of the drug at the tumor site in vivo. This finding contrasts with the findings of some observational studies, which indicated that LEV use during radiochemotherapy was associated with improved OS (Roh et al., 2020)– (Kim et al., 2015) In this study, no significant difference was found between the group that took LEV throughout the entire period of radiochemotherapy and the group that partially used the drug or did not use it at all. Additionally, taking LEV, regardless of its duration, during the radiochemotherapy period vs. not taking it did not show statistical significance. The results of this study are consistent with other research that did not observe any association between LEV use and increased survival. (Happold et al., 2016), (Chen et al., 2022)However, these studies did not take into account the duration of LEV intake in relation to the radiochemotherapy period, whether it was used throughout the entire period of adjuvant therapy or only partially (Knudsen-Baas et al., 2016).

### 4.2 Effect of using Lacosamide on OS

To date, no studies have been conducted to evaluate the potential impact of LAC on survival in patients with GBM, despite its widespread use in the treatment of BTRE. There is some evidence, however, suggesting a possible antitumor effect of LAC based on an in vitro study (Rizzo et al., 2017). This study showed that LAC could modulate the expression of miRNAs and increase the levels of p53, but only if p53 is in wild-type form. The inhibitory effect of LAC on cell cycle progression and migration in these cell lines was observed at concentrations between 300 and 800 μM (Rizzo et al., 2017). Interestingly, better results were obtained with drug concentrations exceeding the doses typically administered for seizure control. Furthermore, the study showed that LAC has the ability to increase intracellular levels of p53, but this effect is limited to cases where p53 is not mutated (Rizzo et al., 2017).

Herein, the survival analysis did not demonstrate any statistically significant differences between the 4 therapy groups. These results contradict those observed in the in vitro experiments for both LEV and LAC. The lack of statistically significant differences in survival could be attributed to the same reasons mentioned earlier regarding LEV. In addition to the potential reduction in drug concentration at the tumor site in vivo, the heterogeneous molecular characteristics of GBM cells and the limited molecular characterization, particularly in terms of the mutational status of p53 and the expression of miRNAs, may also explain the discrepancy between our results and the expectations set by in vitro studies.

### 4.3 Control of seizure recurrence

Our analysis of seizure control demonstrated that LAC, compared to LEV therapy, has a 2.57-fold increase in the IRR of seizure recurrence, regardless of other confounding factors such as preoperative seizure history, MGMT promoter methylation status, and lesion location.

During the study period (2015–2021), especially in the earlier years, LAC was not commonly used as a first-line treatment but rather as an add-on therapy for patients who did not achieve satisfactory seizure control in monotherapy with other ASMs. Even if in our cohort for control of seizure recurrence analysis we considered only patients in monotherapy with one ASM, thus excluding add-ons or switching between them, LAC could be administered in cases where the tumor could be considered more epileptogenic or the seizures more severe. The observed lower effectiveness of LAC compared to LEV should be toned down and viewed in the context of this potential selection bias.

Taking into account the effects of covariates in the present analysis, the hypermethylation status of the MGMT promoter may have a protective effect against seizure recurrence in the postoperative period. This finding appears to contradict recent studies that associate low MGMT expression levels with an increased likelihood of experiencing more seizure episodes after surgery compared to cases with an unmethylated MGMT promoter. The less aggressive behavior of tumors with MGMT promoter hypermethylation, characterized by expansive rather than infiltrative growth, may contribute to a higher risk of seizure recurrence in the postoperative period. [26,808,114 mgmt does not protect against seizures]. Our results could be explained by considering the response of GBM cells with hypermethylated MGMT promoters to TMZ chemotherapy. Lower levels of the MGMT enzyme, which repairs alkylating agent-induced damage (including TMZ), are associated with a better response to chemotherapy (Hegi et al., 2005). Thus, reduced transcription of the MGMT gene due to hypermethylation leads to increased tumor sensitivity to TMZ (Weller et al., 2015). The protective effect observed in our study may be attributed to the improved response of these tumors to adjuvant therapy, resulting in reduced tumor volume and subsequently decreasing the epileptogenic effect of any residual tumor portions after surgery. However, since other potentially confounding molecular factors were not included in our model, the interpretation of the methylation status of the MGMT promoter as a possible preventive factor for postoperative seizure occurrence should be approached with caution and further investigated in future clinical studies.

### 4.4 Limitations

Apart from the retrospective nature of the study, the main limitation is that the sample size was reduced by the need to exclude confounding factors, such as taking other ASMs, and patients who did not receive standard radio- and chemotherapy treatment. In addition, the lack of molecular analysis of some genes, such as p53, may have underestimated the effect of LEV and LAC on survival in these subgroups of patients, even if observed in in-vitro studies. Lastly, the fact that lacosamide was not the first line ASM for the majority of the study observation period, could lead to a selection bias when comparing the effectiveness of the two drugs on seizure control.

## 5 Conclusions

This study did not find any statistically significant differences in OS among GBM patients who used LEV vs. LAC during adjuvant radiochemotherapy protocol. For the first time, this study investigated the impact of LAC on survival based on recent in vitro studies suggesting its potential antitumoral effect. Nevertheless, this research contributes additional information to the existing body of literature on the survival effects of LEV, which has thus far yielded conflicting results. Given the molecular heterogeneity of GBM, in vitro studies show partial benefits of ASMs in certain molecular subtypes, such as those with wild-type p53 or high MGMT expression levels (specifically for LEV). Therefore, additional clinical trials are needed to evaluate the antitumoral efficacy of these drugs.

## Funding

This research did not receive any specific grant from funding agencies in the public, commercial, or not-for-profit sectors.

## Disclosures

The authors have no personal, financial, or institutional interest in any of the drugs, materials, or devices described in this article.
